# Clinical response of advanced cancer patients to cellular immunotherapy and intensity-modulated radiation therapy

**DOI:** 10.4161/onci.26381

**Published:** 2013-10-17

**Authors:** Kenichiro Hasumi, Yukimasa Aoki, Ryuko Wantanabe, Dean L Mann

**Affiliations:** 1 Hasumi International Research Foundation; Tokyo Research Center; Tokyo, Japan; 2 Department of Pathology; University of Maryland School of Medicine; Baltimore, MD USA

**Keywords:** activated T cells, cancer vaccine, cytotoxic T lymphocytes, immature dendritic cells, intratumoral injection

## Abstract

Patients afflicted with advanced cancers were treated with the intratumoral injection of autologous immature dendritic cells (iDCs) followed by activated T-cell infusion and intensity-modulated radiation therapy (IMRT). A second round of iDCs and activated T cells was then administered to patients after the last radiation cycle. This complete regimen was repeated for new and recurring lesions after 6 weeks of follow-up. One year post therapy, outcome analyses were performed to evaluate treatment efficacy. Patients were grouped according to both the number and size of tumors and clinical parameters at treatment initiation, including recurrent disease after standard cancer therapy, Stage IV disease, and no prior therapy. Irrespective of prior treatment status, 23/37 patients with ≤ 5 neoplastic lesions that were ≤ 3 cm in diameter achieved complete responses (CRs), and 5/37 exhibited partial responses (PRs). Among 130 individuals harboring larger and more numerous lesions, CRs were observed in 7/74 patients that had received prior SCT and in 2/56 previously untreated patients. Some patients manifested immune responses including an increase in CD8^+^CD56^+^ lymphocytes among circulating mononuclear cells in the course of treatment. To prospectively explore the therapeutic use of these cells, CD8^+^ cells were isolated from patients that had been treated with cellular immunotherapy and IMRT, expanded in vitro, and injected into recurrent metastatic sites in 13 individuals who underwent the same immunoradiotherapeutic regimens but failed to respond. CRs were achieved in 34 of 58 of such recurrent lesions while PRs in 17 of 58. These data support the expanded use of immunoradiotherapy in advanced cancer patients exhibiting progressive disease.

## Introduction

Currently, few treatment options are available for patients with recurrent and advanced stage malignancies that fail to respond to conventional therapeutic modalities. One approach has been to conduct clinical trials to develop new drugs targeting a particular type of cancer. These drugs are often, if not routinely, pushed to a dosage nearing toxic levels in order to achieve an antitumor response. In many cancer centers, an alternative approach with considerably less toxicity (and thus morbidity) is being explored, an approach designed to stimulate antitumor immune responses in the host. These studies are progressing along with advances in our understanding of the complexities of the immune response and the potential to manipulate these responses to constrain malignant disease. One of the important recent advances in this area is the recognition that dendritic cells (DCs) are obligate contributors to the elicitation of efficient immune responses.^1^ Thus, a number of clinical trials have been initiated to test the use of DCs as antigen-presenting vehicles activate immunity and generate effective antitumor immune responses.^2^^-^^6^

It is well established that DC-based vaccines, comprising DCs expanded and loaded with tumor-associated antigens (TAAs) ex vivo, can effectively induce cellular immunity in vitro as well as in vivo. However, so far limited clinical benefits have been achieved even upon the elicitation of a demonstrable immune response to TAAs. There are numerous reasons that may account for such a lack of clinical efficacy, including the well-documented immunosuppressive nature of the tumor microenvironment.^7^ Indeed, it has been demonstrated that the lack of antitumor effector responses correlates with the presence of regulatory T cells and myeloid-derived suppressor cells in the tumor microenvironment.^8^^-^^10^ Another factor that may negatively influence the clinical efficacy of immunotherapy is the imbalance between tumor burden and number of cytotoxic effector cells that can be delivered by passive transfer or stimulated by active immunization.

A variation on this approach that may offer improved efficacy is to combine immunotherapy with conventional treatments designed to decrease tumor burden and control or diminish the activity of tumor-infiltrating immunosuppressive cells.^11^^-^^14^ One such conventional therapy, irradiation, has been shown not only to kill malignant cells, thereby freeing potential antigenic constituents and stimulating the release of pro-inflammatory cytokines that enhance DC function, but also to alter the immunoregulatory tumor microenvironment.^15^^-^^20^

The clinical protocol described herein was developed to explore the efficacy of combining an immunotherapeutic approach with radiation to treat patients with advanced malignancies. The immunotherapeutic components of this regimen comprised autologous immature dendritic cells (iDCs) generated from the monocytes isolated from peripheral blood mononuclear cells (PBMC), as well as patient-derived, activated T cells. These were prepared from the residual T cells present among monocyte-depleted PBMCs and expanded in the presence of high-dose interleukin (IL)-2 in flasks coated with anti-CD3 antibodies.^21^ Recurrent primary and metastatic tumors, identified by positron emission tomography-CT (PET-CT) were directly injected with iDCs in media containing a cytokine cocktail and Keyhole limpet hemocycanin (KLH), followed by infusion of activated T cells. Intensity-modulated radiation therapy (IMRT) was delivered over a number of days in divided doses to the sites of iDC injection. Several days after the last radiation cycles, treated lesions were re-injected with iDCs and additional activated T cells were infused. Patients were evaluated 6 weeks after this treatment cycle and new or recurring lesions were treated with the same regimen.

We previously reported the safety and feasibility of this protocol in a small scale clinical trial involving 26 patients affected by a variety of advanced cancers.^21^ We now report the results of this immunoradiotherapeutic approach in a larger cohort of 167 patients who have been observed for 1 y (to date) after the last treatment cycle. Clinical responses were monitored by PET-CT using Response Evaluation Criteria in Solid Tumors (RECSIT), revealing patients who manifested complete responses (CRs) and partial responses (PRs), particularly among those individuals with few (< 5) and small (< 3 cm each) tumors.

In the course of treatment, various parameters were monitored to detect changes that might reflect the elicitation of an immune response. We observed an increase in CD8^+^CD56^+^ cells among circulating lymphocytes. Cells with this phenotype have been reported by others to have dual cytotoxic activities as mediated by natural killer (NK) cells as well as by CD8^+^ cytotoxic T cells.^22^^,^^23^ To explore the therapeutic potential of such candidate antitumor cells, autologous CD8^+^ T cells were isolated from the peripheral blood of 13 patients who manifested recurrent disease in spite of the immunoradiotherapeutic protocol described above. After IL-2-mediated expansion in vitro, autologous CD8^+^ T cells were injected into 58 metastatic lesions, eliciting a CR in 59% and a PR in 29% of the cases.

Taken together, our data demonstrate the potential of combining immunotherapy with a conventional treatment modality to effectively treat cancer patients with advanced disease.

## Results

### Clinical responses of patients receiving combinatorial immunotherapy with iDCs and activated T cells followed by IMRT

The therapeutic regimen employed in this study, including the intratumoral administration of autologous iDCs, the infusion of patient-derived, IL-2 activated T cells and IMRT, is described in the Materials and Methods section and is illustrated in Figure 1. The outcome of such an immunoradiotherapeutic regimen among 167 patients affected by distinct neoplasms at 1-y follow-up is summarized in Table 1. One single exception in the therapeutic regimen applied involved one glioblastoma patient who received an intra-carotid infusion of iDCs following radiation (the case is summarized together with the PET-CT results in Figure S1). An attempt was made to list all the variables that might influence treatment outcome, although this proved to be a formidable task, especially relative to treatment specifications that in many instances could be determined only based on each patient’s memory. We grouped patients on the basis of several concrete factors that were expected to influence treatment outcome: tumor burden (volume and number of lesions), disease recurrence after standard therapy, and clinical presentation with Stage IV disease coupled to no previous anticancer treatment. Overall, 29 patients dropped out of the study upon their request. One year following the last cycle of treatment, among 37 patients initially presenting with recurrent or stage IV disease and bearing ≤ 5 neoplastic lesions that were ≤ 3 cm in diameter, 23 exhibited a CR (62.2%), 5 manifested a PR (13.5%), 2 had stable disease (SD, 5.4%) and 7 displayed progressive disease (PD; 18.9%). Among 74 patients with larger and more numerous tumors who presented with recurrent disease, 7 exhibited a CR (9.4%), 3 displayed a PR (4.1%), 2 had SD (2.7%) and 62 (83.8%) had progressed. Finally, among 56 patients that presented with stage IV disease and large tumor burden, 2 were found to exhibit a CR (3.6%), 2 manifested a PR (3.6%) and the remaining 52 exhibited PD (92.9%).

**Figure:**
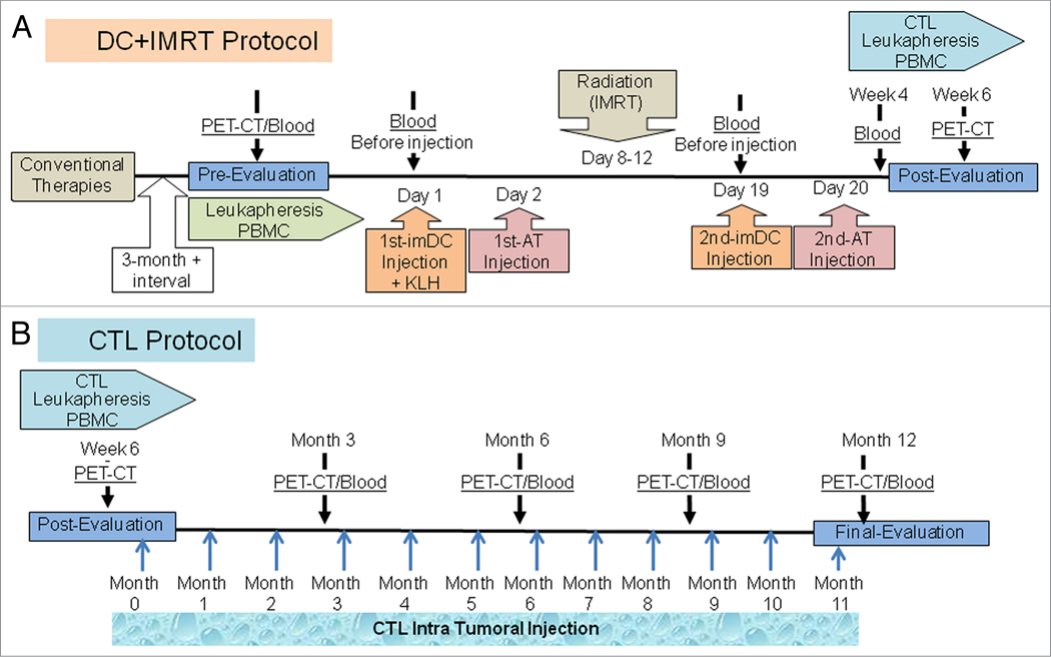
**Figure 1.** Immunoradiotherapeutic protocol employed in this study. (**A**) DC-IMRT protocol: sequence of administration of immature dendritic cells (iDCs), activated T cells and intensity modulated radiation therapy (IMRT) followed by the re-administration of the iDCs and activated T cells. (**B**) CTL protocol: isolation of cells for the generation of cytotoxic T lymphocytes (CTLs) and timing of subsequent intratumoral injection (blue arrows).

**Table:** **Table 1.** Patient outcome at 1-y follow-up evaluation after treatment with immunotherapy and IMRT

(1) Recurrent + Stage IV					
**CR**	**PR**	**SD**	**PD**	**CR + PR**	**Drop Out**
23/37	5/37	2/37	7/37	28/37	5
62.20%	13.50%	5.40%	18.90%	75.70%	
**(2) Out of protocol patients (Recurrent)**					
**CR**	**PR**	**SD**	**PD**	**CR + PR**	**Drop Out**
7/74	3/74	2/74	62/74	10/74	17
9.40%	4.10%	2.70%	83.80%	13.50%	
**(3) Out of protocol patients (Stage IV)**					
**CR**	**PR**	**SD**	**PD**	**CR + PR**	**Drop Out**
2/56	2/56	0/56	52/56	4/56	7
3.60%	3.60%	0%	92.80%	7.20%	

Patients were grouped based on tumor size, number and disease status. (1) Patients with ≤ 5 tumor sites, ≤ 3cm size and with recurrent and/or stage IV disease. (2) Patients with more numerous and larger tumors than 1 with recurrent disease. (3) Patients with more numerous and larger tumors than 1 with Stage IV disease. **Abbreviations:** CR, complete response; PD, progressive disease; PR, partial response, SD, stable disease.

A more detailed description of patients bearing a relatively low number of small tumors is presented in Table 2. Fourteen different types of cancer were treated in these 37 patients. The small number of patients affected by the same type of neoplasm does not allow for the assessment of the efficacy of this approach in specific oncological conditions. However, our data clearly demonstrate the ability of this immunoradiotherapeutic approach to provide clinical benefits to patients with a relatively low tumor burden, regardless of tumor type. Examples of similar responses to treatment as evinced by pre- and post-treatment PET-CT have been previously reported.^21^^,^^24^ Additional data are provided in Figure S1.

**Table:** **Table 2.** One-year evaluation of 37 cases treated over a period of 5 y

Diagnosis	CR	PR	SD	PD	CR + PR
Breast	5	2		1	7/8
Lung	3	1		1	4/5
Ovarian	3				3/3
NPC	3			1 (Bleeding)	3/4
Kidney	2			1	2/3
Gastric	2				2/2
Colorectal		2	1	1 (perforation)	2/4
Uterus (Endometrium)	1			1	1/2
Uterus (Cervix)				1	0/1
Prostate	1				1/1
Lymphoma	1				1/1
Brain (Glioblastoma)	1				1/1
Thymoma	1				1/1
HCC			1		0/1

Patients in this group presented with ≤ 5 tumor sites, ≤ 3 cm in size and with recurrent and/or stage IV disease. Abbreviations: CR, complete response; PD, progressive disease; PR, partial response, SD, stable disease.

### Immunoradiotherapy increases the abundance of circulating CD8^+^CD56^+^ cells

While monitoring patients subjected to the immunotherapeutic regimen described above for changes in immunological parameters, we observed an increase in circulating CD56^+^ cells, including a cell subset that also expressed the T-cell marker CD8. These CD3^+^CD8^+^CD56^+^ T cells were present among circulating PBMCs. An example of the expression profile of these markers, as evinced by flow cytometry, on the PBMCs of a gastric cancer patient is shown in Figure 2. In parallel studies, we determined that CD8^+^CD56^+^ immune cells are capable of killing autologous and allogeneic cancer cells by mechanisms that are known to mediate the cytotoxicity of natural killer (NK) cells as well as through T-cell receptor (TRC) engagement (manuscript in preparation). In light of these observations, we naturally hypothesized that patient-derived immune cells displaying this cytotoxic T lymphocyte (CTL) phenotype and function could be used therapeutically. This concept was experimentally tested using the protocol illustrated in Figure 1, according to which neoplastic lesions that failed to respond to immunoradiotherapy were injected with autologous PBMC-derived CTLs that had been expanded in vitro in the presence of IL-2.

**Figure:**
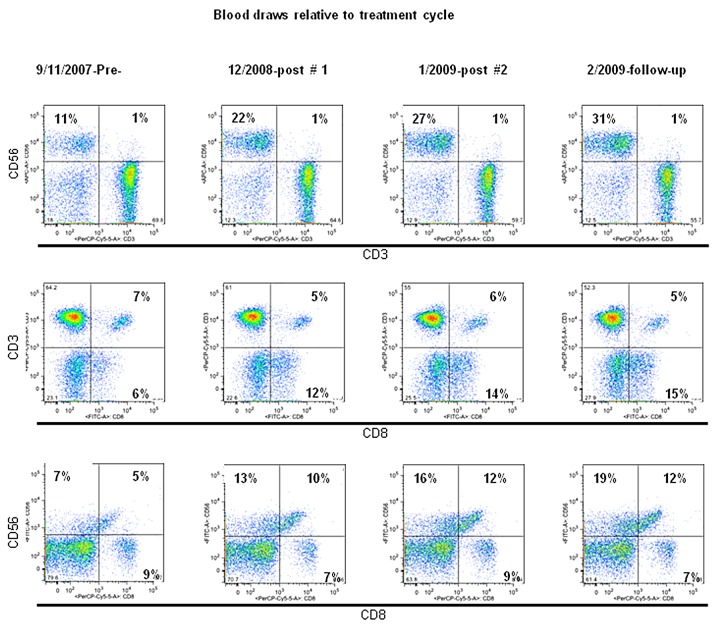
**Figure 2.** Expression of CD3, CD8 and CD56 among peripheral blood mononuclear cells isolated before and after immunoradiotherapy. Representative dot plots of peripheral blood mononuclear cells (PBMCs) obtained from a patient with metastatic gastric cancer before (first panel in each row) and after (1st round, 2nd round, and 1-y follow-up, as indicated) the immunoradiotherapeutic regimen described in Figure 1A, upon staining with anti-CD3, anti-CD8 and anti-CD56 antibodies.

### Therapeutic responses to the intratumoral injection of CTLs

CTLs were prepared from the PBMCs of patients who manifested incomplete responses to previous cycles of iDCs, activated T cells, and IMRT, as evinced by new lesions identified by PET-CT. IL-2-expanded lymphocytes expressing combinations CD3^+^CD8^+^CD56^+^ surface markers (5x10^8^ cells) were injected into tumors with a diameter < 10 mm. Autologous CTLs from 13 patients with a variety of cancer types were injected into a total of 58 neoplastic lesions (involving various organs) over an 8-mo period. Anatomical sites and local clinical responses after 1 or 2 CTL injections are reported in Table 3. Three months after the first CTL injection, the majority of the injected sites manifested objective responses, as documented by PET-CT, including 34/58 (59%) CRs, 17/58 (29%) PRs, 6/58 (10%) SDs, in contrast to disease progression in 1/58 (2%) lesions. A second round of treatment for lesions manifesting PR or SD resulted in 50% CRs and PRs at these sites (Table 4).

**Table:** **Table 3.** Clinical response after CTL injection

Diagnosis, Pathology	Site Injected	Response after 1st Inj	Response after 2nd Inj	Site Injected	Response after 1st Inj	Response after 2nd Inj	Site Injected	Response after 1st Inj	Response after 2nd Inj	Site Injected	Response after 1st Inj	Response after 2nd Inj
Breast, Adeno Ca.	Ax LN	CR		Carinal LN	CR		pleura	CR				
Lung, Adeno Ca.	Sub carinal LN	PR	CR	lung Primary	PR	CR	hiler LN	PR	PR	pleura	SD	PR
	lung	PR	PR	lung	PR	CR						
Thyroid, Thymoma	Thyroid	PR	CR									
Ovarian	Carinal LN	PR		Iliac LN (1)	PR		Iliac LN (2)	PR		Iliac LN (3)	PR	
	SCLN	CR		Ax LN (1)	CR		Ax LN (2)	CR		Ax LN	PR	
Ovarian, Clear cell Ca.	Botallo LN	CR										
Breast, Invasive ductal ca.	Inter Thoracic	CR										
Lung, Aden Ca. suspected	hilar LN	CR		pleura	PR		lung S9	CR				
Ovarian, Serous Adenoca.	pelvic LN (1)	CR		pelvic LN (2)	CR							
Breast, Papillotubular Ca	Ilium	CR		Ilium	CR		Lumbar soft tissue	SD		acetabulum	CR	
	Inter Thoracic	CR		Inter Thoracic	SD		Thoracic soft tissue	CR		Sternum	CR	
Colorectal, Adeno Ca.	iliac LN	PR	PR	lung S6	PD							
Colorectal Adeno Ca.	Liver	CR		Cervical LN1	CR		Cervical LN4	CR		Thoracic soft tissue	CR	
	Cervical LN	PR		Cervical LN5	CR		Cervical LN7	CR		lung S1	PR	
Uterine cervix, Invasive Squamous cell Ca.	PALN (1)	CR		PALN (2)	SD		Caval LN (1)	SD		PALN (3)	CR	
	PALN (4)	CR		Caval LN (2)	CR		iliac LN	CR		lung S3	PR	
Uterine cervix,	caval LN	PR	PR	iliac LN	CR		iliac LN	SD	CR	SCLN (1)	CR	
	SCLN (2)	CR		SCLN (3)	CR		SCLN (4)	CR				

Abbreviations: CR, complete response with disappearance of tumor at treated site; PR, partial response with over 30% reduction in treated sites; SD, stable disease little to no change; PD, progressive disease increase in size of targeted lesion and/or new lesions

**Table:** **Table 4.** Response in tumors injected with cytotoxic T lymphocytes as assed by PET-CT

First Injection			Second Injection		
CR	34/58	59%			
PR	17/58	29%	CR	4/8	50%
			PR	4/8	50%
			SD	-	-
			PD	-	-
SD	6/58	10%	CR	1/2	50%
			PR	1/2	50%
			SD	-	-
			PD	-	-
PD	1/58	2%			

Abbreviations: CR, complete response with disappearance of tumor at treated site; PR, partial response with over 30% reduction in treated sites; SD, stable disease little to no change; PD, progressive disease increase in size of targeted lesion and/or new lesions

A representative example of the therapeutic response observed after CTL injection is shown in Figure 3. This patient was a 55-y-old male harboring a recurrent lung cancer with involvement of multiple lymph nodes and pleural metastases. CTL were injected into 4 (pleural and lymph node) metastatic sites. At the 3-mo post-treatment evaluation, treated lesions manifested objective responses, ranging from PRs to CRs. A new lesion that appeared in the right supraclavicular lymph node was injected and a subsequent follow-up at 3 mo post-treated revealed CRs at all sites. Additional examples of objective responses after the intratumoral injection of CTLs are provided in Figure S1.

**Figure:**
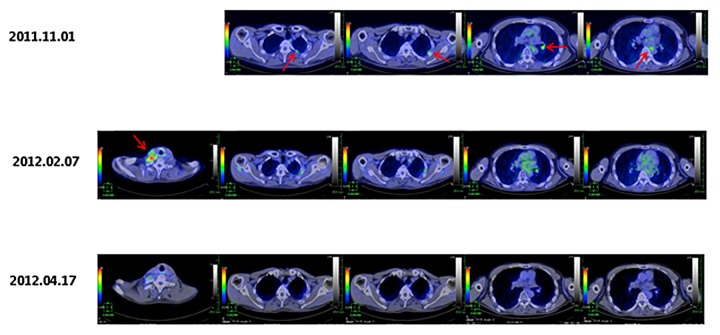
**Figure 3.** Therapeutic responses to the intratumoral injection of therapy-primed cytotoxic T lymphocytes. Representative PET-CT images of metastatic lung cancer lesions (arrows) injected with autologous cytotoxic T lymphocytes (CTLs) generated ex vivo from CD8^+^ T cells isolated from the peripheral blood mononuclear cells upon the failure of immunoradiotherapy. The chronological progression is indicated by date and demonstrates tumor regression.

## Discussion

We developed a protocol combining immunotherapy and radiation to treat patients with a myriad of advanced cancers involving various organs and anatomic sites. Our objective was to establish a therapeutic framework that could be applied to recurrent and/or metastatic cancers appearing at multiple sites. This study was presaged by preclinical studies conducted by us and others that suggested the potential therapeutic efficacy of this combinatorial treatment modality and providing rationale for clinical applications. PET-CT was primarily used to evaluate clinical responses in the course of treatment. Other objective criteria have also been used to evaluate the effectiveness of this anticancer therapy. These data have been reported in a prior publication and include the levels of circulating tumor markers over the course of treatment.^21^

As previously reported, we observed little to no toxicity in response to this immunoradiotherapeutic regimen.^21^^,^^24^ Additionally and of critical clinical relevance, our treatment induced responses not only in iDC-injected and IMRT-treated tumors, but also in distant non-treated tumors, at least in some patients. These observations suggest that our therapeutic protocol stimulated a systemic adaptive immune response against malignant cells. This can only be demonstrated ex vivo by testing the immunoreactivity of CTLs against available autologous tumor cell lines or, alternatively, by assaying serum biomarkers if the CTL-targeted antigens are known. Cell lines were established from tumor biopsies from several of the patients in the study presented herein. We have tested the cytolytic capacity of such patient-derived PBMCs and found that the CD8^+^CD56^+^ cells accumulating upon treatment are able to kill autologous (but not allogeneic) cancer cells (manuscript in preparation). Previously, we examined serum samples from some of our patients for the presence of antibodies against TAAs. Antibodies targeting mesothelin, a TAA expressed by some malignancies, were identified in post-treatment serum samples.^21^ In this prior study as well as in the work presented here, KLH was co-administered with iDCs to serve as a surrogate marker for an immunological response to antigens in the tumor environment. Antibody reactivity toward this neo-antigen was previously observed in patient sera, indicating that either resident antigen-presenting cells or injected iDCs were capable of taking up KLH and eliciting an adaptive immune response.^21^ Further examination of serum samples from the large cohort of patients reported herein are underway to detect the presence of KLH-targeting antibodies and validate this finding.

The ability of intratumorally injected DCs to solicit an antitumor immune response has been reported by others. Chi and colleagues observed signs of an immune response in hepatocellular carcinoma patients who received an intratumoral injection of iDCs combined with radiation, including antibodies against α-fetoprotein and increased NK-cell activity.^12^ Furthermore, this combinatorial treatment was found to enhance the efficacy of infused cytotoxic T cells in a murine melanoma model.^20^

It has been well established that cytokines and chemokines play a fundamental role in the ability of iDCs to both engulf antigens from the microenvironment and migrate to lymphoid organs, the crucial immunological tissues where adaptive immune responses are elicit.^25^^-^^28^ To increase the amount of pro-inflammatory cytokines present at the injected sites, a battery of cytokines generated by CD3/CD28-activated T cells in vitro was co-administered with iDCs. This combination of cytokines and chemokines has been previously shown to exhibit a robust adjuvant activity when administered with standard vaccines.^29^ Beyond potentially exert an antineoplastic cytolytic activity, activated T cells also produce pro-inflammatory cytokines that are known to stimulate immune responses. Moreover, activated T cells express ligands for DC co-stimulatory molecules, hence engaging in a paracrine interaction that enhances immune responses to antigens engulfed by DCs and regulates the character of the T-cell response generated.^24^

Radiation has been reported to stimulate the antitumor immune responses elicited by immunotherapy in pre-clinical studies as well as in several clinical trials.^15^^-^^20^ In addition to potentially stimulating the antitumor activity of cytolytic cells, irradiation can induce an inflammatory response encompassing the release of potent immune mediators that augment innate and adaptive immune responses. Radiation induces the apoptotic and necrotic demise of malignant cells, underpinning the release of cellular components including TAAs that can be acquired by iDCs that are injected within neoplastic lesions. Radiation therapy may also exert immunostimulatory effects by dampening the immunosuppressive tumor microenvironment, thereby affecting both the afferent and efferent arms of an immune response.^17^ Here, we employed IMRT to limit the injury of tissues adjacent to the tumor target, an important consideration given that many of these patients had received radiation as a component of their past treatment.^30^^,^^31^

In addition to the well-documented immunosuppressive environment of most solid tumors, another major impediment to successful cancer immunotherapy is the imbalance between tumor burden and the capacity of the host to develop an immune response that suffices to eliminate the bulk of malignant cells. The importance of tumor burden in the clinical response to immunotherapy is well illustrated in our study, as the most favorable responses developed in patients with limited numbers of lesions that were 3 cm or less in size. This appeared to be an important factor contributing to clinical outcome regardless of tumor origin and irrespective of whether or not the patient had received and failed prior treatment or presented with advanced disease and had not received conventional therapy.

In order to gain insights into that nature of the immune response generated by our immunoradiotherapeutic regimen, we monitored the distribution of subsets of lymphocytes in the peripheral blood with a particular focus on the different cell populations that could be mediate cytolytic activity. In many patients, we observed an increased number of circulating CD8^+^CD56^+^ lymphocytes upon treatment. Other investigators have previously reported that immune cells with this phenotype have cytolytic activity against cancer cells and that CD8^+^ cells co-expressing CD56 may be generated in vitro by culturing PBMCs in media containing the powerful cytokine IL-2.^32^^-^^34^ There have been numerous clinical trials based on tumor-infiltrating T lymphocytes and/or peripheral blood-derived (so-called “lymphokine-activated killer”) cells, serving as proof-of-principal that activated T cells can provide therapeutic benefits to cancer patients. When peripheral blood cells are cultured in the presence of high concentrations of IL-2, a population of immune cells that have been designated cytokine-induced killer cells arises. These culture conditions promote the expression of CD56 on CD8^+^ T cells and render them capable of killing cancer cells through both receptor-ligand interactions that generally mediate the cytotoxic activity of NK cells and TCR-dependent pathways resulting in direct target cell lysis. A comparison of the cytolytic capacity of cytokine-induced killer cells, lymphokine-activated killer cells and tumor-infiltrating lymphocytes has been provided by Jakel and colleagues.^34^ Our observations and those of others prompted us to explore the possibility that CD8^+^CD56^+^ cells may exert therapeutic activity. To test this hypothesis, we isolated CD8^+^ T cells from the peripheral blood of patients treated with the immunoradiotherapeutic regimen described above and expanded them in the presence of anti-CD3 monoclonal antibodies and high concentrations of IL-2 in vitro. These cells were then directly injected into metastatic lesions that had reoccurred after the initial round of treatment. A substantial proportion of these lesions manifested CRs 3 mo after treatment and an additional CTL injection further increased the number of CRs and PRs. Our data demonstrate the antitumor efficacy of this subset of immune cells and support further investigation of their potential application for the treatment of metastatic cancers.

In summary, we report here the treatment of patients with advanced cancers with a combinatorial approach integrating immunotherapy and local irradiation. The rationale for our immunoradiotherapeutic approach came from prior observations by us and others, including results from both in vitro and in vivo studies. The most favorable outcome was observed in patients with advanced recurrent and metastatic cancers harboring low a tumor burden. Immune cell populations that developed in the course of treatment course were shown to have exert antitumor activity expanded in vitro and re-injected into new or recurrent lesions. The intratumoral injection of these CTL into new metastases of limited size proved to be quite effective in patients that had recurrent disease following the administration of iDCs coupled to IMRT. It is important to emphasize that little to no toxicity was observed in patients undergoing this treatment. We shall continue to monitor the clinical and immunological responses of the advanced cancer patients treated with our immunoradiotherapeutic regimen with the objective of increasing our knowledge in order to develop more efficacious therapies for such patients in the future.

## Materials and Methods

### Patients

All patients with either recurrent disease after standard treatment or stage IV disease and no prior treatment were self-referred to either the Shukokai Clinic or the Tokyo Clinic and Research Institute. Patients gave written informed consent for the procedures and protocols after detailed explanation and discussion, according to the Declaration of Helsinki. The consent forms and procedures were approved by the Institutional Review Boards of the respective institutions.

### Treatment protocol

An overview of the therapeutic protocol is provided in Figure 1. Prior to treatment, the extent of disease and location of metastasis were established by PET-CT. Patients underwent leukapheresis to obtain monocytes for differentiation into iDCs, and the monocyte-depleted, T-cell enriched fraction was used to prepare activated T cells. iDCs were combined with lymphocyte-conditioned media (LCM), a multi-cytokine based adjuvant^29^ and KLH. This mixture was divided into aliquots bearing equivalent cell numbers based on the number of sites to be injected, the volume of each aliquot was adjusted to < 2 mL with PBS, and individual lesions were injected under PET-CT guidance. Activated T cells were infused the following day. Approximately 7 d later, the injected neoplastic lesions received IMRT in divided doses. Seven days following the last IMRT cycle, neoplastic lesions were again injected with iDC suspended in LCM (without KLH) and activated T cells were infused the next day. Blood samples were obtained prior to protocol initiation and periodically thereafter to monitor changes in the levels of tumor markers and the development of anti-KLH antibodies. PET-CT exams were repeated 6 weeks following the treatment cycle and at approximately 3-mo intervals thereafter. Four weeks after the second injection of iDCs, the patients underwent a second leukapheresis to obtain the population of CTLs to be expanded in vitro. Expanded cells were stored in liquid nitrogen until use. Patients who developed new lesions received intratumoral injection of these cells.

### Collection and isolation of PBMCs

Leukapheresis were performed on a COBE Spectra blood separator (Gambro KK), using the program for collection of mononuclear cell population (version 7.1). Anticoagulant citrate dextrose solution (ACD-A; ratio of 12:1, Baxter) was used for anticoagulation. The inlet rates were 40–60 mL/min with a collection rate of 1 mL/min and a separation factor of 700. PBMCs were isolated by gradient separation, suspended at 1.5x10^8^ cells/mL and cryopreserved (−80°C) in AIM-V^®^ medium (Gibco, Invitrogen) containing 10% dimethylsulfoxide (DMSO). Prior to use, iDCs, activated T cells and CTLs were thawed in a 37°C water bath, washed twice in AIM-V^®^ medium and re-suspended at the concentrations required for injection.

### Preparation of iDCs

Thawed PBMCs (approximately 6 x 10^8^ cells) were suspended in 20 mL AIM-V^®^ medium and distributed in 5 mL aliquots to four T75 cm^2^ polystyrene flasks containing 10 mL of AIM-V^®^ medium. After 2 h of incubation at 37°C, non-adherent (monocyte-depleted) cells were removed and destined to activated T-cell preparation (see below). Fifteen mL of DC growth medium, that is, AIM-V^®^ medium supplemented with 800 IU/mL granulocyte macrophage colony-stimulating factor (GM-SCF; from CellGenix) and 500 U/mL IL-4 (BD PharMingen), was added to each flask containing adherent cells. Flasks were incubated at 37°C with 5% CO_2_. On day 3, growth media was refreshed. On day 7, iDCs were harvested and either prepared for injection, or re-suspended in AIM-V^®^ freezing medium containing 20% autologous serum and 10% DMSO, then cryopreserved in BICELL containers (Nihon Freezer Co.).

### Preparation of activated T cells

Approximately 6–9x10^8^ non-adherent T cells were collected from cultures of PBMCs that had been depleted by adhering monocytes to generate iDCs. The remaining cells, including T cells, were cultured in T225 cm^2^ flasks coated with 5 µg/mL anti-CD3 antibody (Orthoclone, OKT3 injection) in 35 mL AIM-V^®^ medium supplemented with 1,000 IU/mL IL-2 and 10% autologous serum. Flasks were incubated for 7 d at 37°C with 5% CO_2_. Three hours prior to harvesting, 1 µg/mL ionomycin (Sigma) was added to the medium. Harvested cells were extensively washed and prepared for infusion or cryopreserved, as described above.

### Preparation of LCM

Detailed protocols to prepare products of activated lymphocytes for use as an adjuvant is described elsewhere.^29^ In brief, lymphocytes were suspended in 50 mL XVIVO 10 medium (Cambrex) containing human T-expander CD3/CD28 Dynabeads (Invitrogen) at a cell: bead ratio of 1:1. This cell suspension was incubated at 37°C in 5% CO_2_ for 2 d. The supernatants were harvested by centrifugation at 300 g for 7 min and stored at 4°C for later use. LCM was injected to patients together with iDCs.

### Preparation of CTLs

Cryopreserved PBMCs were suspended in 20 mL AIM-V^®^ medium supplemented with 10% autologous serum at a concentration of 5x10^6^ cells/mL and incubated at 37°C in 5% CO_2_ for 24 h. After washing, cells were re-suspended in isolation buffer and CD8^+^ cells were isolated using Dynabeads^®^Untouched^TM^ Human CD8 T Cells kit (Invitrogen Dynal), according to the manufacturer’s instruction. After isolation, CD8^+^ cells were washed twice and re-suspended in AIM-V^®^ medium supplemented with 2000 IU/mL IL-2 and 5% autologous serum. CD8^+^ cells were adjusted to a concentration of 1x10^6^ cells/mL and cultured for 5 d in flasks coated with OKT3 antibodies (Janssen Pharmaceutical KK), harvested and cultured for an additional 25 d in uncoated flasks. Finally, cells were harvested and either prepared for injection or cryopreserved at −80°C for subsequent use.

### Sterility and endotoxin testing

The presence of microbial contaminants was tested by incubating cultured cells on agar at 37°C with subsequent inspection for bacterial growth. Endotoxin levels (< 0.5 EU/mL) were determined using a commercially available chromogenic endotoxin assay kit (Toxicolor system LS-50M, Seikagaku Corp.), according to the manufacturer’s instructions. Only sterile and endotoxin-free preparations were used clinically.

### Characterization of iDCs, activated T cells and expanded CD8^+^ T cells

A standard fluorescence cytometry labeling protocol was used to determine the surface phenotype of iDCs using fluorochrome-conjugated monoclonal antibodies against CD11c, CD14, CD40, CD80, CD83, CD86, and HLA-DR (BD PharMingen). Activated T cells were evaluated for CD3, CD4, CD8, CD25, CD56, and CD154 (CD40L) expression after culture. CTLs were evaluated for CD3, CD4, CD8, and CD56 expression following culture. Data were analyzed using the Cell Quest software package (BD Biosciences). The immunophenotype of iDCs, activated T cells and CTLs is summarized in Table 5.

**Table:** **Table 5.** Expression of surface markers on cell populations employed in treatment

Dendritic Cells		Activated T-Cells		Cytotoxic T Lymphocyte	
Marker	Percentage	Marker	Percentage	Marker	Percentage
CD11c	87 ± 12	CD3+CD4+	51 ± 17	CD3+	88 ± 15
CD14	32 ± 28	CD3+CD8+	38 ± 14	CD4+	2 ± 2
HLA-DR	69 ± 23	CD3+CD56+	31 ± 16	CD8+	95 ± 7
CD40	39 ± 27	CD3-CD56+	8 ± 5	CD3+CD4+	1 ± 2
CD80	30 ± 18	CD62L	16 ± 7	CD3+CD8+	49 ± 17
CD83	22 ± 15	CD154	25 ± 13	CD56+	31 ± 14
CD86	70 ± 23	CD25	88 ± 18	CD56+CD3+	29 ± 14
CD3	3 ± 3			CD56+CD3-	2 ± 1

### Preparation of cells for injection

iDCs and activated T cells were thawed in a 37°C water bath approximately 1 h prior to injection. One mL of AIM-V^®^ medium was added to each thawed vial. Vials were then incubated for 2 min at 22 ± 3°C (room temperature), transferred to 50 mL of medium and centrifuged at 300 g for 7 min to remove DMSO. iDCs were suspended in 10% LCM, then prepared in one microtube and placed on ice for transport. Activated T cells were suspended in 100 mL of normal saline containing 1.25% albumin and 10% LCM and infused i.v.. CTLs for intratumoral injection were prepared as described above.

### Radiation

IMRT was delivered to each iDC-injected neoplastic lesions. The total dose, delivered in fractions, was determined to be such that the biologically effective dose was 72 Gy, according to the linear quadratic model. Each fraction was optimized such that the radiation-induced late toxicity to surrounding normal tissue would be equal to or less than Grade 2 based on the National Cancer Institute Common Terminology Criteria (NCI-CTC) version 2.0 system.

### Toxicity monitoring

Patients were monitored for signs and symptoms of adverse reactions to therapy, including vital signs (temperature, blood pressure, pulse, and respiration), diaphoresis, arthralgia, and swelling at injection sites, as well as blood chemistry and hematological analyses. Other signs and symptoms that might be associated with radiation or the injection of cells by insertion of a needle through various organs in order to reach metastatic lesions were also monitored. Blood tests showed no changes in blood cell counts or biochemistry, as determined by hemogram and biochemical data. Basically all treatments were well tolerated with no adverse reactions with the exception of 9 patients that developed transient temperature elevation (> 38°C) after IMRT.

### Evaluation of clinical responses

PET-CT imaging was used to assess clinical response to treatment based on the Response Evaluation Criteria in Solid Tumors (RECIST) guidelines. The first exam was conducted 6 weeks after the end of the last treatment cycle with periodic follow-up PET-CT exams. Responses to treatment were defined as follows: complete response (CR), complete disappearance of tumor at the treated site and no new lesions at distant sites; partial response (PR), 30% reduction in the size of targeted lesions; progressive disease (PD), increase in size of targeted lesion and/or appearance of new lesions; and stable disease (SD), little to no changes in size of targeted lesion and no new lesions.

## Supplementary Material

Additional material

## Abbreviations:

AX: axillary
CR: complete response
CTL: cytotoxic T lymphocyte
iDC: immature dendritic cell
HCC: hepatocellular carcinoma
IMRT: intensity-modulated radiation therapy
KLH: Keyhole limpet hemocyanin
LCM: lymphocyte conditioned media
NK: natural killer
NPT: no prior therapy
PALN: peri-aortic lymph node
PMBC: peripheral blood mononuclear cell
PD: progressive disease
PET-CT: positron emission tomography-computed tomography
PR: partial response
SCT: standard cancer therapy
SD: stable disease
SCLN: subclavicular lymph node
TAA: tumor-associated antigen
