# Supplementary material for: Clinical response of advanced cancer patients to cellular immunotherapy and intensity-modulated radiation therapy
Source: Oncoimmunology. 2013 Oct 17;2(10):e26381. doi: 10.4161/onci.26381 (PMC3858384; doi:10.4161/onci.26381)
Supplement: Additional material [file onci-2-e26381-s01.pdf]

Landes Bioscience www.landesbioscience.com

Supplemental Material to:

Kenichiro Hasumi, Yukimasa Aoki, Ryuko Watanabe, and Dean L Mann

Clinical response of advanced cancer patients to cellular immunotherapy and intensity-modulated radiation therapy

OncoImmunology 2013; 2(10)
http://dx.doi.org/10.4161/onci.26381

http://www.landesbioscience.com/journals/oncoimmunology/article/26381/

SUPPLEMENTARY DATA

Patient with Glioblastoma

Glioblastoma after Surgery and Radiation

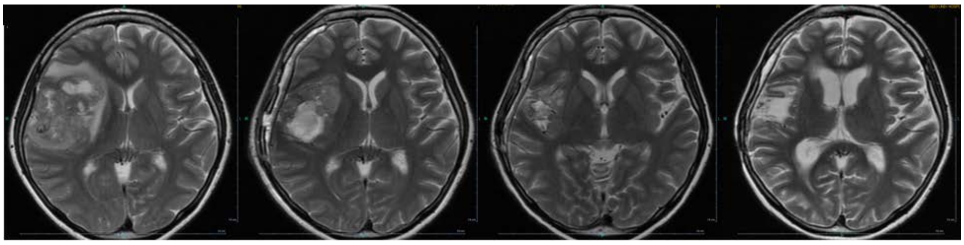

Before surgery 2008.07.30	After surgery 2008.08.29	After radiation, Before iDC, interarterial 2008.10.10	Follow -up CR 2010.12.09

This patient was diagnosed with glioblastoma in 2008. The tumor was treated by surgical resection followed by radiation. Immature DC, prepared from PBMC were injected via bilateral carotid arteries at approximate 3 month intervals over the following 3 years. Serial PET-CT radiographs obtained over the course of disease and treatment are shown in this figure.

Patients treated with combination immunotherapy and radiation

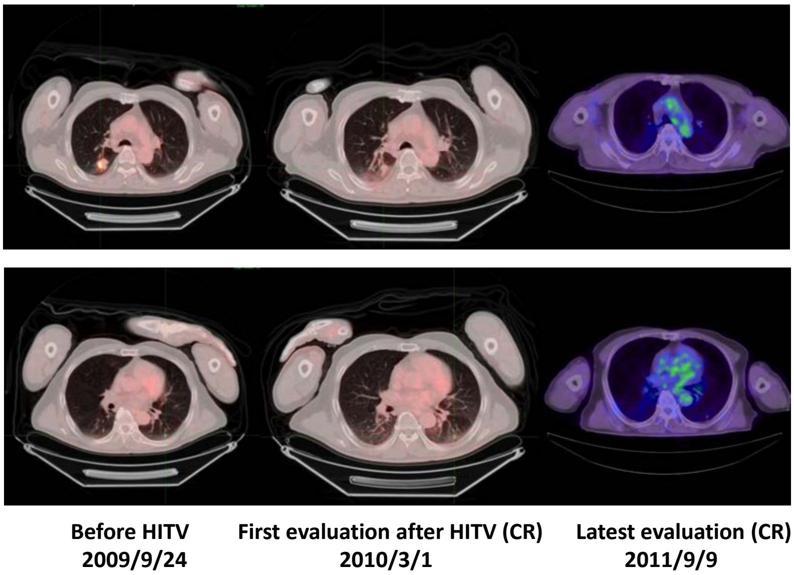

Figure 1A This patient was diagnosed with right lung cancer and brain metastasis (stage IV) that were surgically removed. Metastatic lesions that were subsequently detected in the left and right lungs were injected with iDC and treated with IMRT. The response to treatment is shown in this figure.

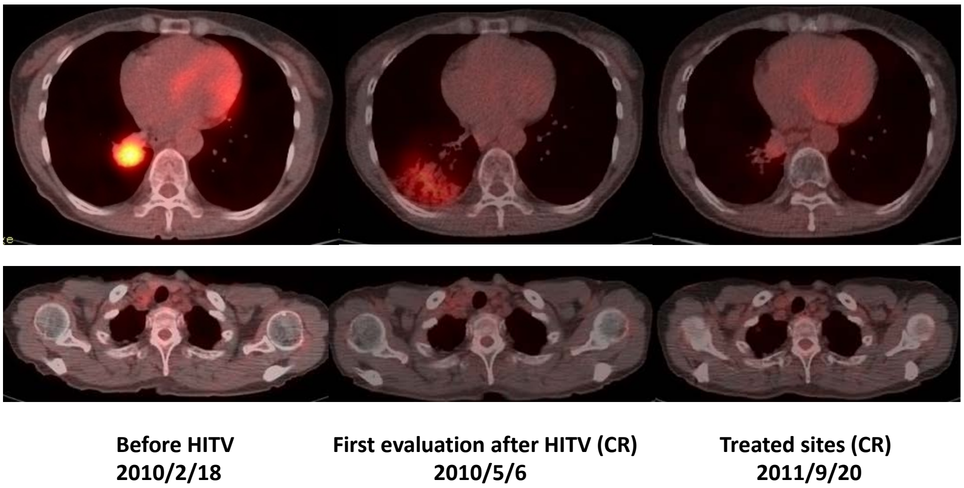

Figure 2A Serial PET-CT images of metastatic lung cancer lesions treated with intra-tumoral injection of iDC followed by IMRT. Radiographs show response of hilar lesions at 1 year follow-up post treatment.

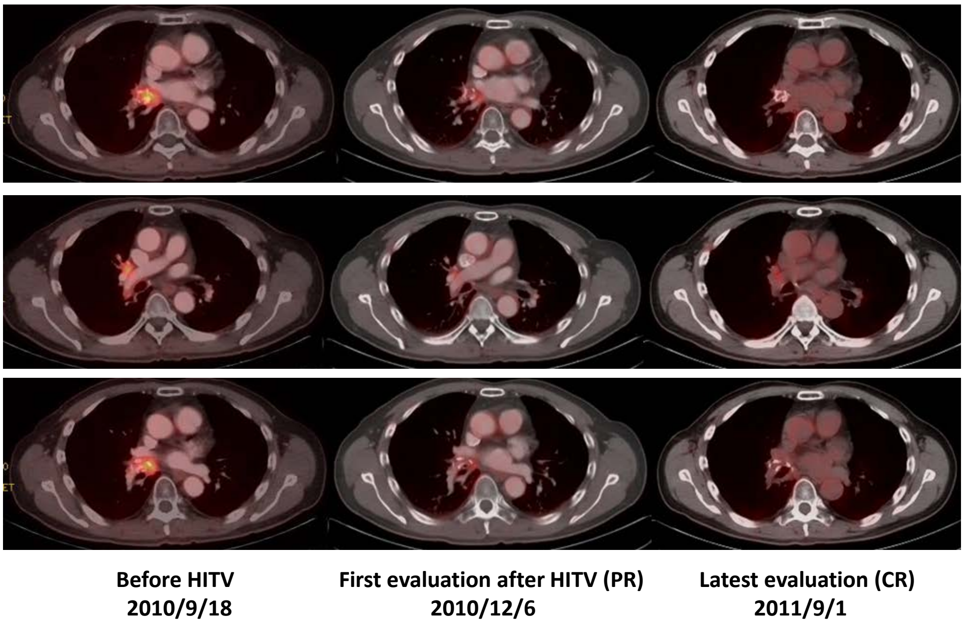

Figure 3A PET–CT images of metastatic lung cancer lesions pre and post treatment with intra-tumoral iDC injection and IMRT.

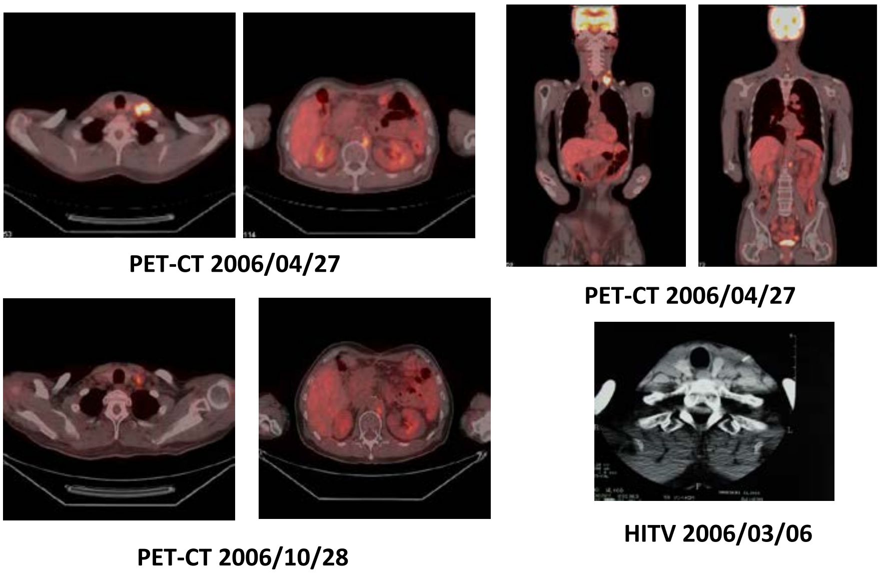

Figure 4A Patient with metastatic gastric carcinoma treated with combination therapy as described in methods section. Follow-up PET-CT images show resolution of treated lesions.

Patients treated with HITV followed by intra-tumoral injection of CTL

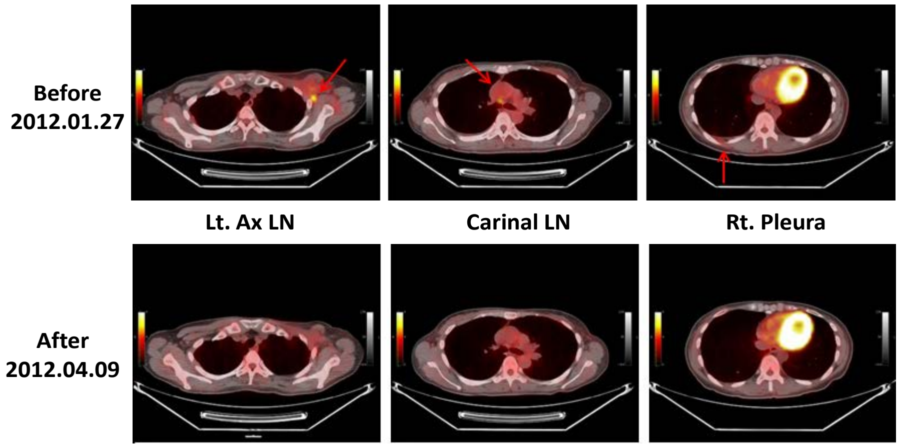

Figure 1B This patient was diagnosed with breast cancer in 2006 and treated with surgical resection followed by chemotherapy. Metastatic disease was subsequently diagnosed and treated with the combination immune-radiadiation therapy described herein Follow-up PET-CT showed complete response in the treated tumors however recurrence was detected in late 2011. Lesions shown in this figure were injected with CTL.

Rt. Iliac LN

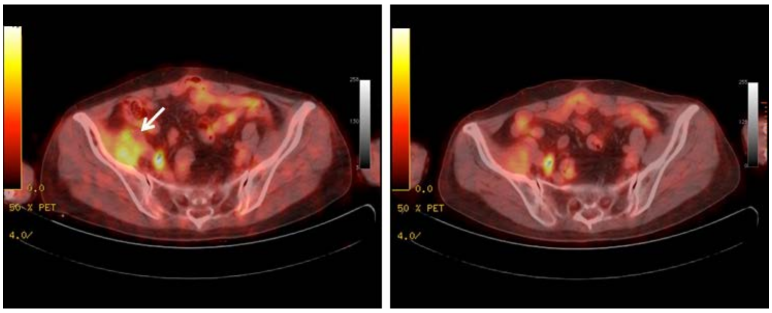

Before / 2012.02.10 **After / 2012.08.01**

Figure 2B This patient, diagnosed with ovarian cancer in 2008, was treated with 6 courses of chemotherapy. Recurrent lesions were initially treated with inter-tumoral injection of iDC and IMRT Subsequent recurrence, indicated by the arrow, was injected with CTL with follow-up response is shown in this figure.

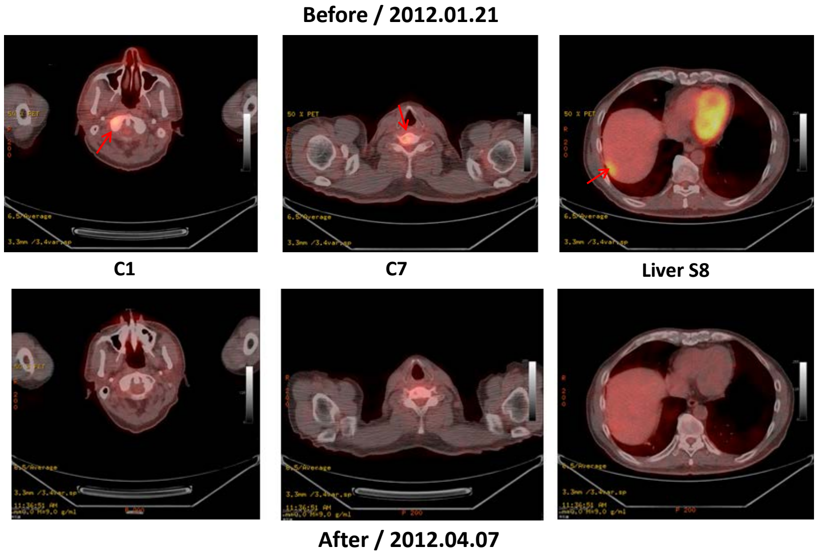

Figure 3B Stage IV rectal cancer was diagnosed in this patient in 2010. The cancer did not respond to chemotherapy. Multiple metastatic lesions were injected with iDC and radiated (IMRT). Subsequent recurrence and response after CTL injection are shown in this figure.

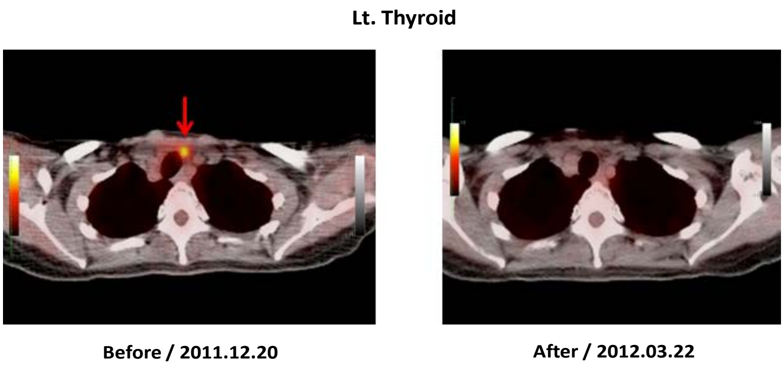

Figure 4B Thyroid cancer was diagnosed in this patient in May 2010. Patient refused surgical intervention and was treated with inter-tumoral iDC injection and IMRT. Progressive disease, documented by follow-up PET-CT, was treated with inter-tumoral CTL injection.
